# Prediction of future healthcare expenses of patients from chest radiographs using deep learning: a pilot study

**DOI:** 10.1038/s41598-022-12551-4

**Published:** 2022-05-18

**Authors:** Jae Ho Sohn, Yixin Chen, Dmytro Lituiev, Jaewon Yang, Karen Ordovas, Dexter Hadley, Thienkhai H. Vu, Benjamin L. Franc, Youngho Seo

**Affiliations:** 1 Department of Radiology and Biomedical Imaging, Center for Intelligent Imaging, University of California, San Francisco (UCSF), 505 Parnassus Ave, San Francisco, CA 94143 USA; 2 Department of Computer Science, University of Illinois Urbana-Champaign, 201 North Goodwin Ave, Urbana, IL 61801-2302 USA; 3 Bakar Institute for Computational Health Science, University of California San Francisco, 505 Parnassus Ave, San Francisco, CA USA; 4 Department of Radiology, Stanford University School of Medicine, 300 Pasteur Dr, Palo Alto, CA USA; 5 University of Central Florida College of Medicine, 6850 Lake Nona Blvd, Orlando, FL 32827 USA

**Keywords:** Biomarkers, Health care, Risk factors, Computer science, Biomedical engineering

## Abstract

Our objective was to develop deep learning models with chest radiograph data to predict healthcare costs and classify top-50% spenders. 21,872 frontal chest radiographs were retrospectively collected from 19,524 patients with at least 1-year spending data. Among the patients, 11,003 patients had 3 years of cost data, and 1678 patients had 5 years of cost data. Model performances were measured with area under the receiver operating characteristic curve (ROC-AUC) for classification of top-50% spenders and Spearman ρ for prediction of healthcare cost. The best model predicting 1-year (N = 21,872) expenditure achieved ROC-AUC of 0.806 [95% CI 0.793–0.819] for top-50% spender classification and ρ of 0.561 [0.536–0.586] for regression. Similarly, for predicting 3-year (N = 12,395) expenditure, ROC-AUC of 0.771 [0.750–0.794] and ρ of 0.524 [0.489–0.559]; for predicting 5-year (N = 1779) expenditure ROC-AUC of 0.729 [0.667–0.729] and ρ of 0.424 [0.324–0.529]. Our deep learning model demonstrated the feasibility of predicting health care expenditure as well as classifying top 50% healthcare spenders at 1, 3, and 5 year(s), implying the feasibility of combining deep learning with information-rich imaging data to uncover hidden associations that may allude to physicians. Such a model can be a starting point of making an accurate budget in reimbursement models in healthcare industries.

## Introduction

Healthcare cost is an important barrier to healthcare access and accounts for a substantial fraction of the national budget. Health care expenses in a society are often distributed according to Pareto-like extreme value distributions, with top 50% spenders account for 97% of the total healthcare expenditures in the United States^1,2^. The top spenders tend to be patients who are sicker and often underserved in society^3^. At an individual patient level, having a reliable cost estimation model can better prepare a patient financially and psychologically. At the hospital and national healthcare policy level, accurate identification and prediction of top healthcare spenders can be a starting point of making an accurate budget and planning appropriately for risk in reimbursement models based on health outcomes.

Recent advances in computer vision models, especially rapid advancements in convolutional neural networks (CNN), has contributed to a variety of applications^5,6^. Deep learning has especially been powerful in identifying mild to moderate associations that humans might not routinely predict or detect in dense images. For example, deep learning has been applied to imaging data to predict and diagnose various diseases such as pneumonia, Alzheimer’s disease, major thoracic diseases, and more^7–10^. In current practice, clinical radiologists might be able to extract information relevant to the clinical question related to the image data, but numerous mild to moderately powered hidden associations, whether clinically relevant or not, may exist within the dense imaging data of the radiograph.

We hypothesize that chest radiographs capture many general health indicators and thus may be utilized, potentially along with information on sex, age, and ZIP code, to predict future medical costs. The chest x-ray radiograph (CXR) is the most commonly performed radiological examination^4^. It allows the examination of the heart, lung, airway, bones of the spine and chest, and blood vessels. Additionally, compared to text or categorical data, a radiograph is a high-dimensional and dense set of data that contains rich and unique information correlated to the patient, similar to that of a profile photo that can be revealing about a person. Ultimately, the prediction harnessed from chest radiographs may be used for proactive preventive care in order to potentially reduce the medical costs for high-risk groups. Therefore, this study aims to investigate the feasibility of developing deep learning models to predict healthcare costs and identify top 50% spenders.

## Methods

### Chest radiograph data

All procedures in this study were approved by the Institutional Review Board of University of California, San Francisco Medical Center, California, USA and performed in accordance with relevant guidelines and regulations. The institutional review board of University of California, San Francisco Medical Center, California, USA waived the need of informed consent for the retrospective use of the CXR data. All participants were non-obstetric adult patients who presented to the emergency department (ED) between July 1, 2012 and November 30, 2016 and received a chest radiograph at the ED or at an outpatient facility on the day of ED presentation. 34,743 frontal chest radiographs were initially identified belonging to 30,823 patients paired with corresponding patient’s age, sex, ZIP code, and cost at UCSF Medical Center within the consequent 1, 3, or 5 year(s). At our institution, frontal chest radiographs for adult patients are typically obtained at 100–120 kVp with automatic exposure control. Source to detector distance is set at 72 inches unless modified for special reasons. Lateral chest radiographs were excluded. More than 90% of the images were acquired on GE and Philips hardware.

### Healthcare spending data

Healthcare spending data was obtained from the cost accounting unit of the institution’s hospital financial department. Total healthcare spending was based on the sum of direct and indirect expenses attributed to patients’ hospital stay, pharmacy, laboratory, imaging, surgeries, and medical consultations over the time period during which they were included in the study. As an outcome, we selected total healthcare expenditure over the subsequent 1, 3, and 5 years.

### Data exclusion

12,869 (37.0%) of 34,743 chest radiographs were excluded during data processing due to some missing patient information (Suppl. Fig. S1). 11,857 (92.1%) of the excluded radiographs did not have information available for their healthcare spending. The rest 1012 (7.9%) of the excluded chest radiographs consisted of 8 with no associated sex, 128 with no associated ZIP codes and 876 whose ZIP code could not be matched to the median income.

### Exploratory data analysis

Pairwise chi-squared test was performed between the cost groups (above and below median expenditure patients) and patient demographic variables such as age groups, sex, geographic area, and race. The effect of demographic variables (sex, age, race, and median income per ZIP code area) and their second-order interaction terms on log_10_-transformed costs was analyzed in multi-factor ANOVA. Missing data analysis was performed to compare 1004 excluded CXRs (not including the 8 with unknown sex) and the 21,872 included CXRs. We analysed association of missingness with demographic variables (sex, geographic area, and race) as well as top-spenders and bottom-spenders using chi-squared test, and association to age using *t*-test (Suppl. Table S1). Among the 21,872 chest radiographs with 1 year expenditure, 9477 (43.3%) are missing expenditure amounts for 3 years or longer and 20,073 (91.8%) are missing expenditure amounts for 5 years. To investigate the contribution of survivorship bias (i.e. patients living longer may have lower medical cost because they were healthier or did not have to pay for end-of-life care), we compared median expenses for patients who dropped out versus those who remained before the 3rd and 5th year after CXR was taken.

### Model architecture

Regression models were developed to predict healthcare expenditures, and binary classification models were developed to predict whether a participant’s healthcare expenditure was in the top 50%. Both regression and classification models were developed in four versions: (T for “Tabular data”) baseline model that relies only on patient sex, age, and ZIP code median income as input, (X for “X-ray”) ResNet^11^ with only CXR images as input, (TX1) separately trained T and X model combined at final stage, and (TX2) modified ResNet trained end-to-end with CXR images, age, sex, and per-ZIP code median income as input. The baseline (T) regression and classification baseline models were gradient boosting regressor^12,13^ and an AdaBoost Classifier^14^ respectively implemented in the Python scikit-learn package with default parameters. The regression and classification CXR-only models (X) were a modified ResNet18 model and a modified ResNet50 model respectively. For combined model TX1 (Suppl. Fig. S2), the raw softmax score or final (regression) output from model (X) were concatenated to categorical data and then processed with model (T) approach to arrive at the output. For combined model TX2 (Suppl. Fig. S3), the neural network architectures from model (X) were modified after the final convolutional layers to allow the concatenation of the categorical data into the neural network model in an end-to-end fashion. See Supplemental Figs. S2, S3 and eAppendix for implementation details.

### Model training

All versions of ResNet were initialized with weights pre-trained on ImageNet^15,16^. For all models, hyperparameters such as learning rate, linear layer dimension, number of linear layers, and others were empirically optimized via random search^17^. After hyperparameter tuning and training, the models were evaluated against the pre-split test set^18^. The training, validation, and test set were split by patient identification numbers to ensure that no two CXR from same patient is represented across multiple datasets. The outputs of the classification model were evaluated using the area under the receiver operator characteristic curve (ROC-AUC), and F1 score. The ROC-AUC of classification models were compared pairwise using DeLong method^19^. The outputs of the regression model were measured using Pearson's R, and Spearman ρ. Confidence intervals (95%) were computed for all statistics. Each training and evaluation were performed for 1 year (21,872 CXR), 3 years (12,395 CXR) and 5 years of expenditure (1779 CXR), respectively. Since 1-year expenditure data was the most complete, all following analysis should be assumed to be based on 1-year expenditure, unless mentioned otherwise.

### Error analysis

For error analysis, we interrogated whether the absolute difference between the true cost value and the predicted cost value is correlated with any of the patient demographic factors. The linear model used percentage differences (|true cost − predicted cost|/true cost) as the dependent variable and patient sex, race, age, median income, and overall true cost as the covariates.

## Results

### Exploratory data analysis

**Figure 1:**
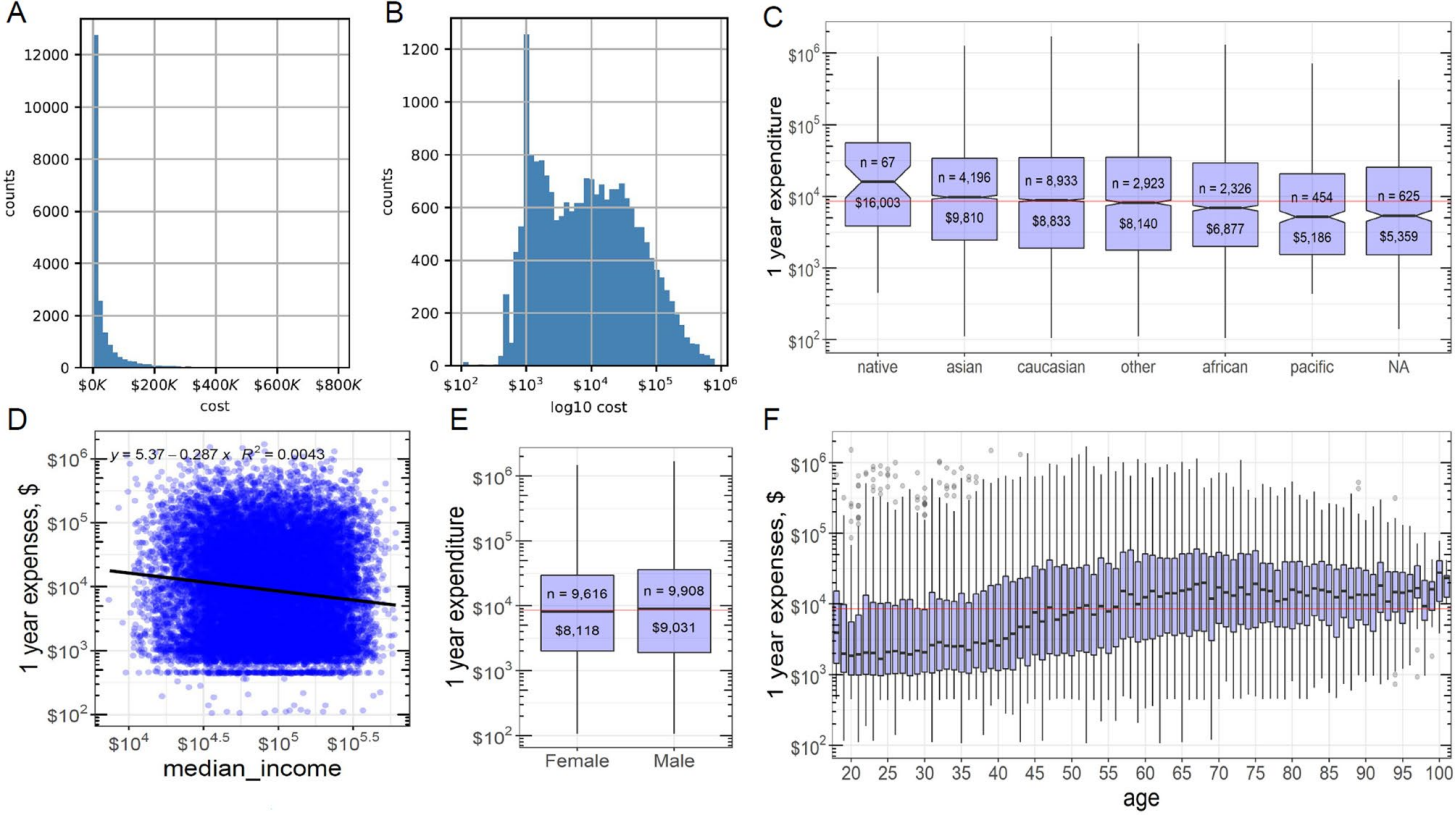
(**A**) Histogram showing the distribution of 1 year total healthcare expenditure. (**B**) Histogram showing log_10_-transformed 1 year of total healthcare expenditure. (**C**) Box plots of 1 year of expenditures for each race variable. The red line shows the population median. (**D**) Scatter plot of median income vs 1-year expenditure. (**E**) Box plots of 1 year expenditures for females and males. The red line shows the population median. (**F**) Box plots of 1 year expenditures for each age number. The red line shows the population median.

After inclusion and exclusion criteria are applied, 21,872 chest radiographs belonging to 19,524 patients with at least 1-year spending data were ultimately used in this study (Table 1). Patients ranged from 18 to 111 years old (56 ± 19.7 SD). Racial representation in our population was not significantly different from the census population in this geographic area (*p* = 0.650 in chi-square)^21^. Among the 19,524 patients, 11,003 patients had 3 years of cost data, and 1678 patients had 5 years of cost data (Table 2). The healthcare expenditures were log_10_-transformed, making data distribution closer to a normal (Fig. 1A,B, Suppl. Fig. S4), with a median of 4.45 ($27,886), and mean ± SD of 4.43 ± 0.82 ($26,953). Significant sources of variation in healthcare expenditures per multi-factor ANOVA included race (*p* = 3e−27, Fig. 1C), median income (*p* = 1.4e−13, Fig. 1D), and age (*p* < 4e−312, Fig. 1F). Sex did not significantly contribute to variance as an independent factor (*p* = 0.6, Fig. 1E), while interation of sex and age (*p* = 9e−06) as well as sex and race (*p* = 0.02) did. Higher ZIP code area median income was associated with lower healthcare expenditures (Pearson *R* = − 0.0537, *p* = 5.4e−14; Spearman ρ = − 0.0544, *p* = 2.4e−14; *n* = 19,612), an association that is robust to outliers and heavy tail effects (see Suppl. eAppendix). The average yearly cost slightly decreased in the course of the study period (2012–2016, adjusted *R*^2^ = 0.0004, regression *p* = 0.0007, see Suppl. Fig. S5 and Suppl. Table S3).

**Table 1:** Patient demographics for prediction of 1-year expenditure.

Characteristics	Count (%)	# below ave cost (%)	# above ave cost (%)
Gender (p = 0.112)*			
Female	9616 (49.3)	5091 (49.8)	4525 (48.7)
Male	9908 (50.7)	5133 (50.2)	4775 (51.3)
Age (p < 0.0001)*			
18–37	4055 (20.8)	2949 (28.9)	1106 (11.9)
38–57	5423 (37.8)	3081 (30.1)	2342 (25.2)
58–76	6212 (31.8)	2634 (25.8)	3578 (38.5)
77–96	3639 (18.6)	1485 (14.5)	2154 (23.1)
97–116	195 (1.0)	75 (0.7)	120 (1.3)
Region (p < 0.0001)*			
Bay area	17,153 (87.9)	9093 (88.9)	8060 (86.7)
Not bay area	2367 (12.1)	1129 (11.1)	1238 (13.3)
Not assigned	4 (0.0)	2 (0.0)	2 (0.0)
Race (p < 0.0001)*			
American Indian or Alaska Native	67 (0.3)	30 (0.3)	37 (0.4)
Asian	4196 (21.5)	2072 (20.3)	2124 (22.8)
Black or African American	2326 (11.9)	1219 (11.9)	1108 (11.9)
Native Hawaiian or other Pacific Islander	454 (2.3)	285 (2.8)	169 (1.8)
Other	2923 (15.0)	1569 (15.3)	1354 (14.6)
Unknown/declined	625 (3.2)	411 (4.0)	214 (2.3)
White or Caucasian	8933 (45.8)	4638 (45.4)	4295 (46.2)

*p-value is comparing the relationship between cost and the corresponding variables using chi-squared test for independence for categorical variables, two-tails at significance level 0.05.

**Table 2:** Performance summary for best models on 1, 3, 5-year(s) expenditure.

Model	1 year	3 years	5 years
Training set size	16,399	9324	1328
Classification ROC-AUC (95% CI) *Corresponding Model*	0.806 (0.793–0.819) *Model TX1*	0.771 (0.750–0.794) *Model X*	0.729 (0.667–0.729) *Model TX2*
Classification F1 (95% CI)	0.779 (0.766–0.791)	0.775 (0.756–0.794)	0.781 (0.736–0.826)
Regression Spearman ρ (95% CI) *Corresponding Model*	0.561 (0.536–0.586) *Model TX2*	0.524 (0.489–0.559) *Model TX2*	0.424 (0.324–0.529) *Model X*
Regression Pearson R (95% CI)*	0.557 (0.532–0.581)	0.523 (0.487–0.558)	0.421 (0.314–0.530)

*CI* confidence interval.

*Pearson R is calculated using log_10_-transformed data.

To test for the contribution of survivorship bias, we compared median expenditures among patients that dropped out and those who did not. One would expect inflated expenditure in a case where drop outs are due to deaths. On the contrary, median expenses among patients that dropped out before year 3 and year 5 were slightly lower than among those who remained (93.26% and 80.38% respectively, *p* < 0.01, Suppl. Table S4). Thus we believe that only a negligible fraction of patients who dropped out of the study died and that patients who remained are more likely to suffer from chronic illnesses. Overall missingness in our dataset was correlated with sex, geographic area, and race (*p* < 0.001 in chi-square test), as well as age (*p* < 0.001, two-sample *t*-test, Suppl. Table S1).

### Classification of top-spenders

**Figure 2:**
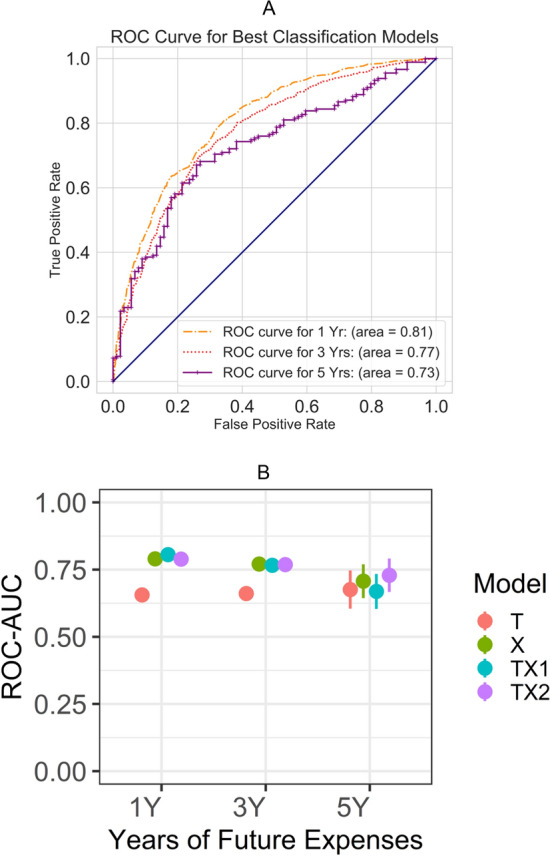
(**A**) ROC Curves for the four classification models. (**B**) Whisker plot of ROC-AUC of top-50% spender classifications within 1, 3, and 5 years. Note that the four models include (T) baseline model that relies only on patient sex, age, and ZIP code median income as input, (X) ResNet with only CXR as input, (TX1) separately trained T and X model combined at final stage, and (TX2) modified ResNet trained end to end with CXR, age, sex, and per-ZIP code median income as input.

Performance results of best models for each time period are shown in Fig. 2. Of the 4 classification models (T, X, TX1, and TX2) trained on 1-year expenditure data (n = 16,399 training CXR), the model TX1, leveraging both demographic data and features extracted by the CNN model, performed best with ROC-AUC of 0.806. For the classification within 3 years (n = 9324), best results were achieved using model X, trained using only CXR as input, with ROC-AUC of 0.771. For the classification within 5 years (n = 1328), best results were achieved using model TX2 (ROC-AUC of 0.729) that combined the inputs of T and X trained end-to-end. Pairwise comparison of the 1-year expenditure results (Suppl. Fig. S6) showed that the ROC-AUC of the classification model T is significantly different from all other models (*p* < 2.2e−16), unlike differences between X and TX1, TX1 and TX2 models (*p*-values > 0.05). We use the TX1 model for subsequent 1-year expenditure analysis.

### Prediction of healthcare costs

**Figure 3:**
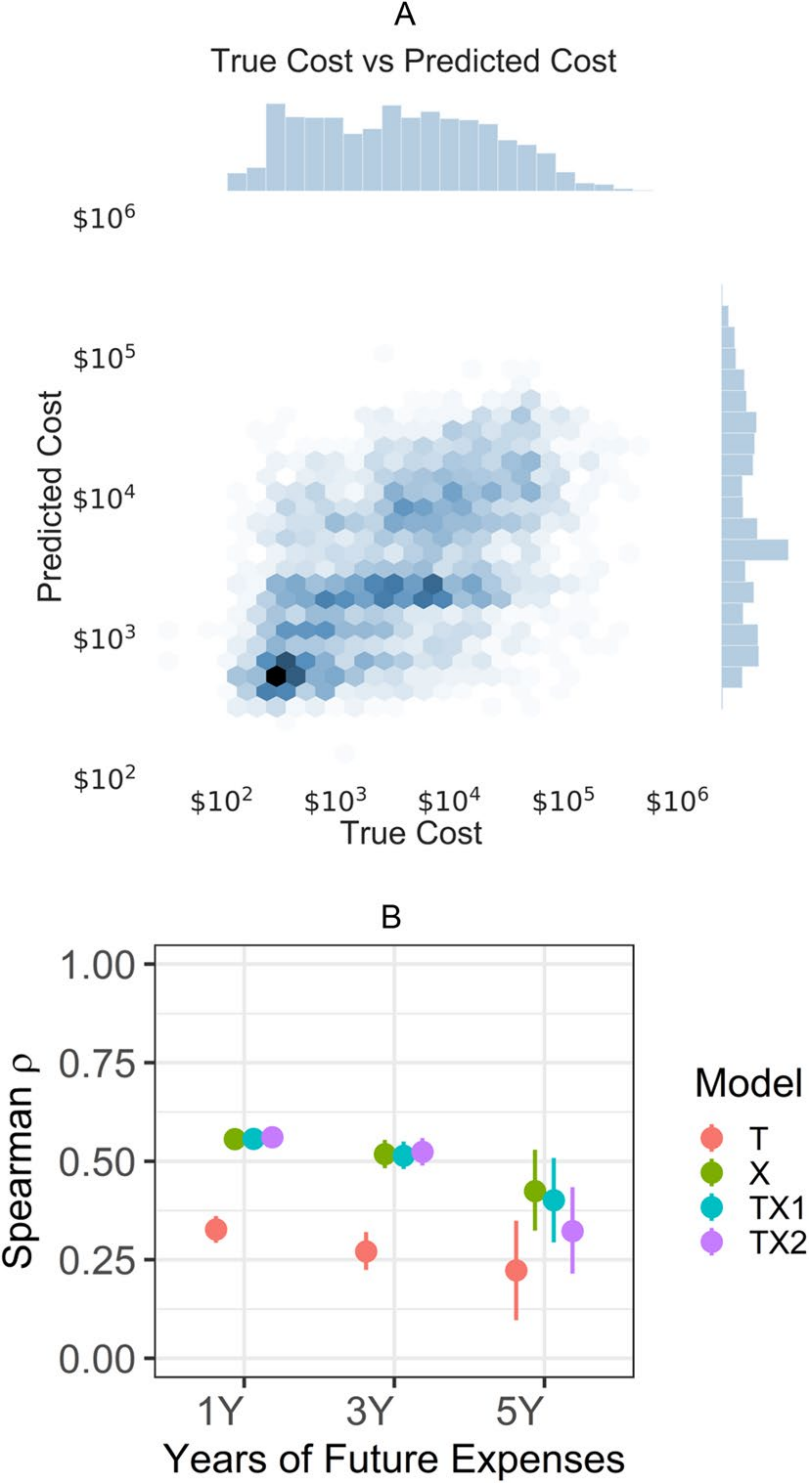
(**A**) Joint distribution hex plot for the TX2 (modified ResNet with all features trained end to end) prediction model. The histogram on x-axis shows log_10_ of the true cost. The histogram on y-axis shows log_10_ of the predicted cost. (**B**) Whisker plot of Spearman ρ for order-of-magnitude prediction within 1, 3, and 5 years.

**Figure 4:**
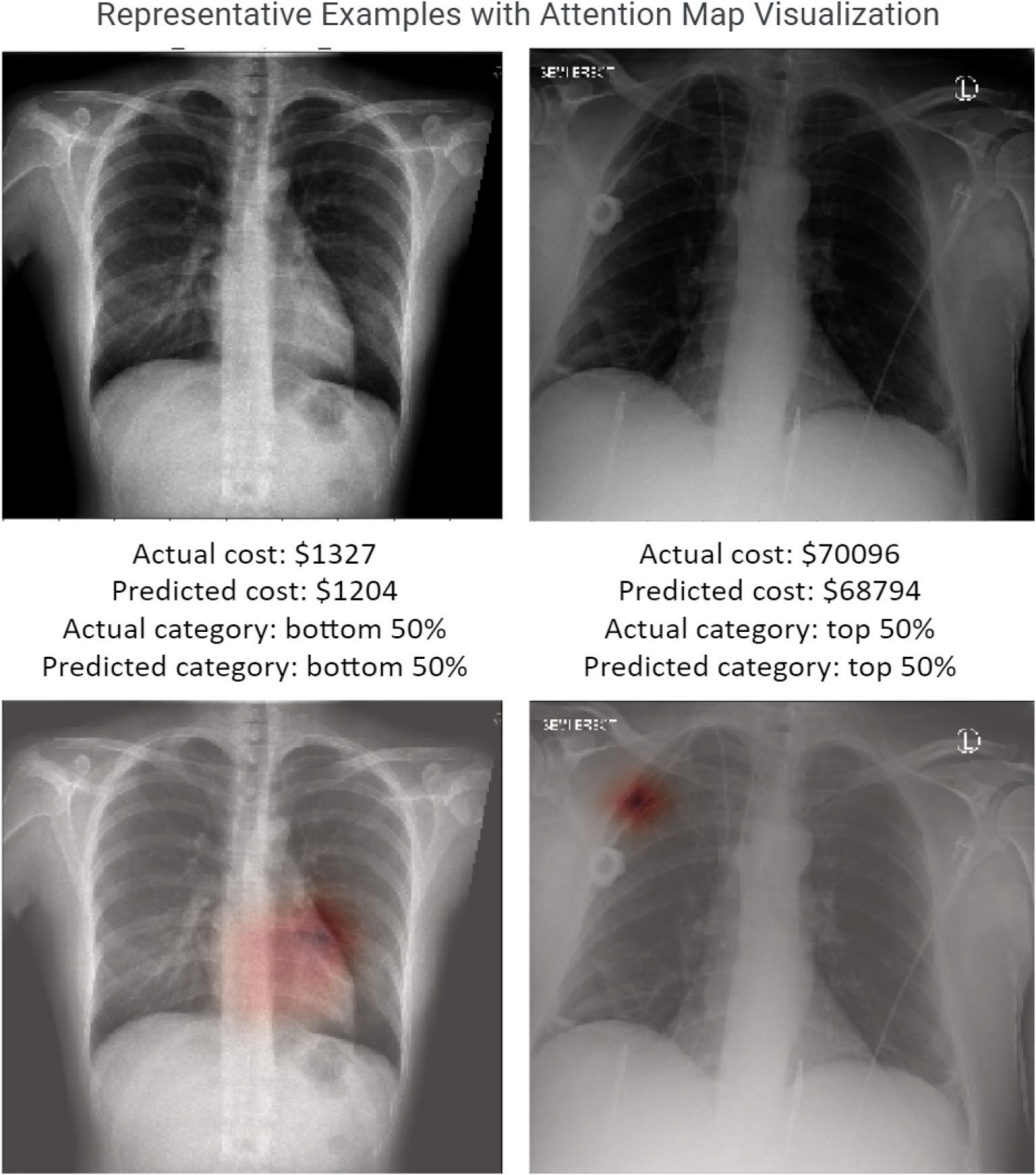
Examples of chest radiographs pairing with Grad-CAM maps of the chest radiographs generated using 1 year data. The top images are the original images in grayscale. The descriptions showed that true and predicted cost values and categories. The bottom images highlighted the regions that are of most importance to the deep learning model’s decision (left: attention on the heart; right: attention on the chest Port-A-Cath). See Suppl. Fig. S6 for CXR with poor performance.

The predicted costs within 1, 3, and 5 year(s) were demonstrated to have a correlation with true costs as illustrated in the joint histogram (Fig. 3A). The prediction model for 1-year costs outperformed the other models for 3- and 5-year costs. The best model for 1-year cost is TX2 that achieved Spearman ρ of 0.561 (*p* = 2e−271) (Fig. 3B). 3-years costs follow with Spearman ρ of 0.524 (*p* = 5e−129) by model TX2. 5-years costs come last with Spearman ρ of 0.424 (*p* = 4e−13) by model X. Examples of chest radiographs pairing with Grad-CAM^20^ maps of the chest radiographs predicted using 1-year expenditure data are shown in Fig. 4. In error analysis, only overall cost (adjusted R^2^ = 0.014, p < 0.01) and ZIP code median income (adjusted R^2^ = 0.040, p < 0.01) were associated with residuals of prediction for model TX1.

## Discussion

We demonstrated the feasibility of predicting healthcare costs and classifying top-50% spenders by using deep learning models based on chest radiographs (CXR) that are widely available in clinics and hospitals. The models were developed to identify patients who are likely to incur high healthcare expenditure and predict their subsequent amount of healthcare spending within 1, 3, and 5 years. Unlike physicians who are trained to identify only a handful of imaging biomarkers known to medical literature, our deep learning algorithm is able to take into account thousands of imaging features of weak to moderate correlations with healthcare spending as presented in the training set. When a CXR is evaluated by the deep learning algorithm, its pixels are aggregated, transformed, and passed through many layers of filters with each layer extracting different lines, angles, patterns, and associations. As those extracted features are then passed upstream to higher-level filters, they are compared to the thousands of CXR that the algorithm was trained on. All these numbers finally converge to the estimated cost^21,22^. Considering that CXR tends to be standardized, deep learning algorithms are trained to be extremely sensitive to details that clinical radiologists may not typically recognize.

From a data scientist perspective, the ability of deep learning algorithms to predict healthcare expenditure from CXR is a testament to the vast amounts of information hidden in imaging data that can be leveraged with data science. The addition of other demographic and clinical variables to the imaging data resulted in minimal improvements to the model, despite the baseline models showing that sex, age, and ZIP code median income are individually associated with healthcare expenditures. This again affirms the presence of rich information within imaging data and the ability of deep learning models to extract them. It is important to note that deep learning algorithms are, at large, approximations based on a large volume of data^23^. The causality for each prediction cannot be definitely deduced^24^. As of any machine learning predictions, it cannot be used as *definitive* proof of a patient’s health or future health expenditure. In addition, there remains ethical concerns as well if the algorithm is used to deny coverage by insurance companies. Nevertheless, the deep learning algorithm can be potentially used by government or insurance companies to identify high-risk individuals and take appropriate actions to secure their health and reduce cost. Such predictions can provide an important starting point in identifying high risk patients to achieve reduction in their healthcare spending and encouraging lifestyle modifications and more intensive medical management to achieve better medical and financial outcomes.

From a clinical perspective, the deep learning algorithm takes into account a combination of demographic factors (age, sex), baseline health factors (weight, bone health), as well as clinical diseases (e.g., enlarged heart, osteophytes, etc.) that are inferred from CXR. For example, having hemodialysis access or enlarged heart from congestive heart failure could be strong indicators of higher healthcare spending predicted by the algorithm. Having replaced hardware or numerous osteophytes could be indicative of older age, which in itself is a predictor of higher healthcare spending as well. While the algorithm does not explicitly give these medical diagnoses when it arrives at its final spending prediction, the algorithm is able to incorporate numerous weak to moderately associated cost predictors in the CXR and assemble them into the final cost predictions.

Additionally, we believe the use case of the model can go beyond simple actuarial calculation purposes. If one accepts that we have shown (A) that our model is predictive of high future healthcare expenses and that it is reasonable to assume (B) that one of the major contributing factors of high future costs is deteriorating health, one must accept that our model indirectly detects signs of deteriorating health with reasonable predictive power. Though such a model would not be able to provide the precise diagnosis, it can sound an alarm to the patient and primary care doctor that the patient will likely have high healthcare spending in the future. Furthermore, our algorithm could be used in outpatient settings to estimate approximate future healthcare costs such that patients, doctors, and insurance companies would have a reliable indicator to consider when making patient treatment and financial decisions. The identified high-risk patients could be subject to more intensive preventive medical interventions and close follow-up visits to modify patient outcomes. The algorithm could also be used to identify patients with CXR that appear normal according to current clinical radiological standards but are still at risk for high medical costs. Similar to most deep learning algorithms, the application of ours can potentially be automatic, fast, scalable, and relatively low cost when compared to other services in the healthcare system.

Several limitations to the study should be noted, primarily related to selection bias inherent in this particular dataset. First, the performance differences between 1, 3, and 5-year models were observed, which can be attributed to both drastic differences in sample size as well as inherent loss of predictive information about the future. The 3-year expenditure model performed slightly worse than the 1-year expenditure model using 56.7% of the sample size. The 5-year model used only 8.1% of the sample size but still achieved reasonably accurate classification and regression results. We believe that with more data the 5-year expenditure model would show even more promise. Second, the development and testing of the model involved data originating from a single hospital system and most lived in the San Francisco Bay Area in the United States healthcare system. The model will likely not generalize to the non-American healthcare system due to the particular structure of healthcare expenses. However, a similar approach can be undertaken to build a new model with any local dataset. Third, missing data (mainly due to missing financial information) constituted 37% of the originally extracted dataset and they were missing not at random. For example, homeless patients may not have had a zip code available. Fourth, patient death information was not available, yet survivorship bias is negligible given that the expenditures among the patients who dropped out do not those in the patients who remained in the study. Fifth, the dataset did not include inpatient cases and portable CXR. Lastly, due to constant changes in healthcare spending because of new treatment and diagnostic testing, the model's actual dollar healthcare cost prediction may become less accurate over time, but the top 50% vs bottom 50% spender categorization would more likely remain relevant.

## Conclusion

We demonstrated the potential of deep learning algorithms to predict 1,3, and 5-years patient healthcare expenditure based on a frontal chest radiograph even in the absence of additional clinical information. This study confirms that radiological imaging indeed contains rich information that may not be routinely extracted by human radiologists but can be analyzed by the power of big data and deep learning. Successfully predicting healthcare expenditure can potentially be an important first step towards improving health policy and medical interventions to address patient care and societal costs.

## Supplementary Information

Supplementary Information.
