# Supplementary material for: Prediction of future healthcare expenses of patients from chest radiographs using deep learning: a pilot study
Source: Sci Rep. 2022 May 18;12:8344. doi: 10.1038/s41598-022-12551-4 (PMC9117267; doi:10.1038/s41598-022-12551-4)
Supplement: Supplementary file 1 — Supplementary Information. [file 41598_2022_12551_MOESM1_ESM.docx]

**Prediction of Future Healthcare Expenses of Patients from Chest Radiographs**

**Using Deep Learning: A Pilot Study**

Jae Ho Sohn, MD, MS^1^*, Yixin Chen, MS^1,2^, Dmytro Lituiev, PhD^3^, Jaewon Yang, PhD^1^, Karen Ordovas, MD^1^, Dexter Hadley, MD, PhD^5^, Thienkhai H. Vu, MD, PhD^1^, Benjamin L. Franc, MD_4_, Youngho Seo, PhD^1^

**Affiliations:**

^1^University of California San Francisco, Department of Radiology and Biomedical Imaging, 505 Parnassus Ave, San Francisco, CA, USA 94143

^2^University of Illinois Urbana-Champaign, Department of Computer Science, 201 North Goodwin Ave, Urbana, IL 61801-2302

^3^University of California San Francisco, Bakar Institute for Computational Health Science, 505 Parnassus Ave, San Francisco, CA, USA

^4^Stanford University School of Medicine, Department of Radiology, 300 Pasteur Dr, Palo Alto, CA, USA

^5^University of Central Florida College of Medicine, 6850 Lake Nona Blvd, Orlando, FL 32827

**Note:** Jae Ho Sohn and Yixin Chen are co-first authors with equal contributions.

***Corresponding Author:**

Jae Ho Sohn, MD, MS

Assistant Professor in Residence

Center for Intelligent Imaging

Radiology and Biomedical Imaging

University of California San Francisco

1-415-476-1000

sohn87@gmail.com

**Supplementary Figures and Tables**


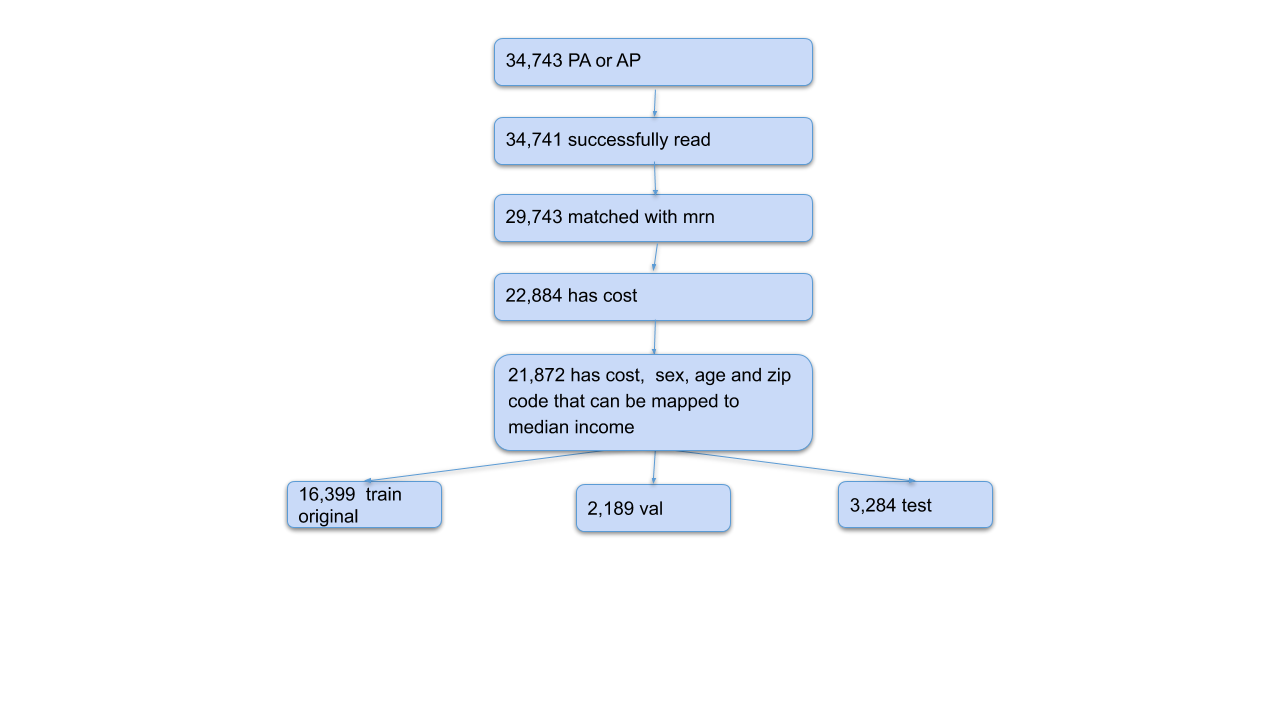


**Figure S1**. Data processing flow chart. Illustration of the number of chest radiographs during each stage of data processing. Note that most are missing due to missing patient identification and financial information.

**
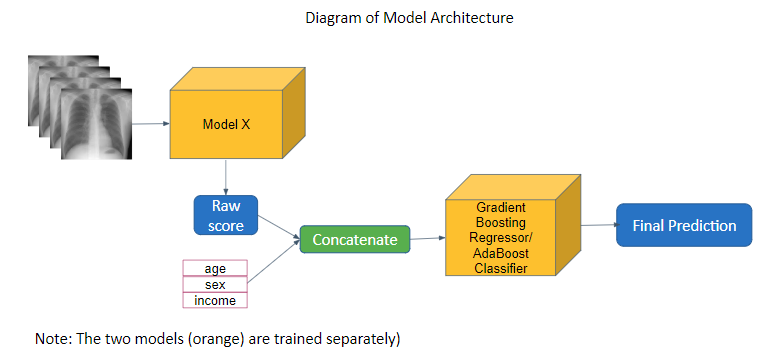
**

**Figure S2**. Model TX1 prediction/classification architecture. For the prediction model, the predictions of model X regressor is concatenated with the additional inputs and feed into a scikit-learn learn default Gradient Boosting Prediction model. For the classification model, the softmax of model X classifier is concatenated with the additional inputs and feed into a scikit-learn learn default Ada Boosting Classification model.


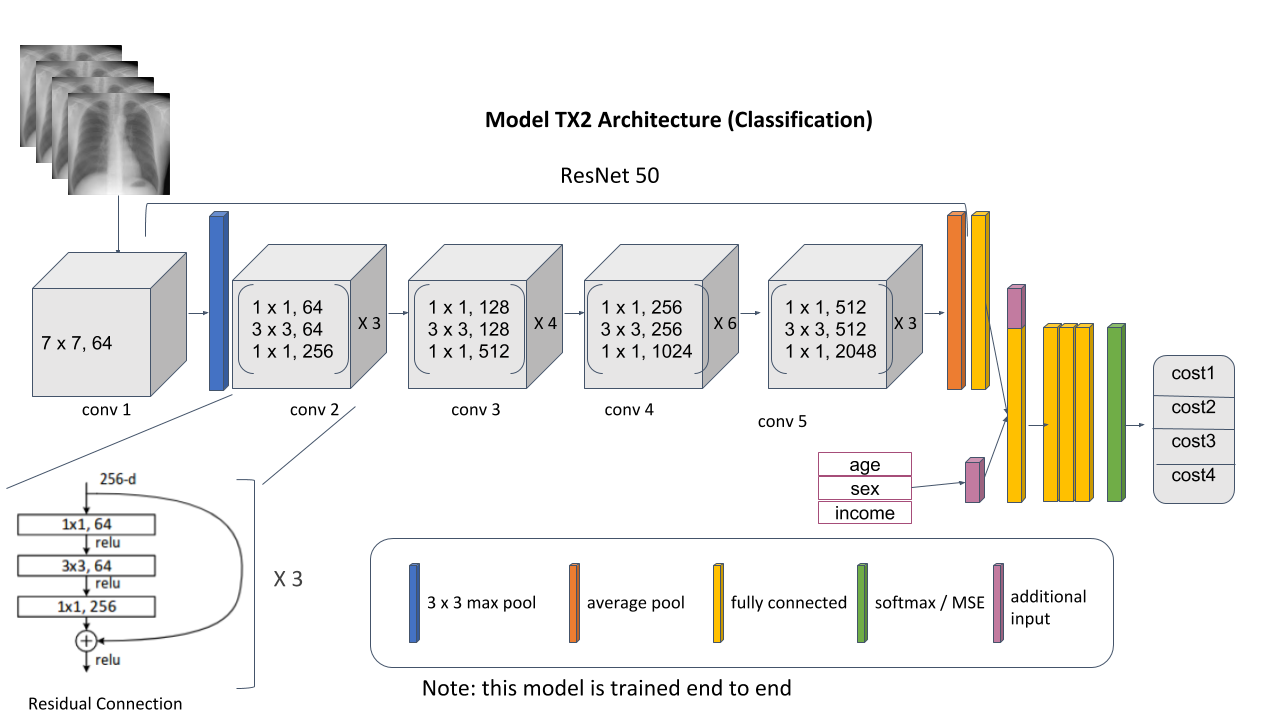


**Figure S3**. Model TX2 prediction/classification architecture. The prediction model is a modified ResNet 18. The original fully connected layer is replaced with a Pytorch Sequential layer consisting of 3 linear layers of output dimensions of 100, 100, and 20 respectively. After the additional input is concatenated, there’s 4 more fully connected layers of output dimensions 10, 10, 10, and 1.The classification model is a modified ResNet 101. The original fully connected layer is replaced with a Pytorch Sequential layer consisting of 2 linear layers of output dimensions of 100, and 20 respectively. After the additional input is concatenated, there’s 5 more fully connected layers of output dimensions 20, 10, 10, 10, and 2. For both models, all linear layers has ReLu layers sandwiched between them.


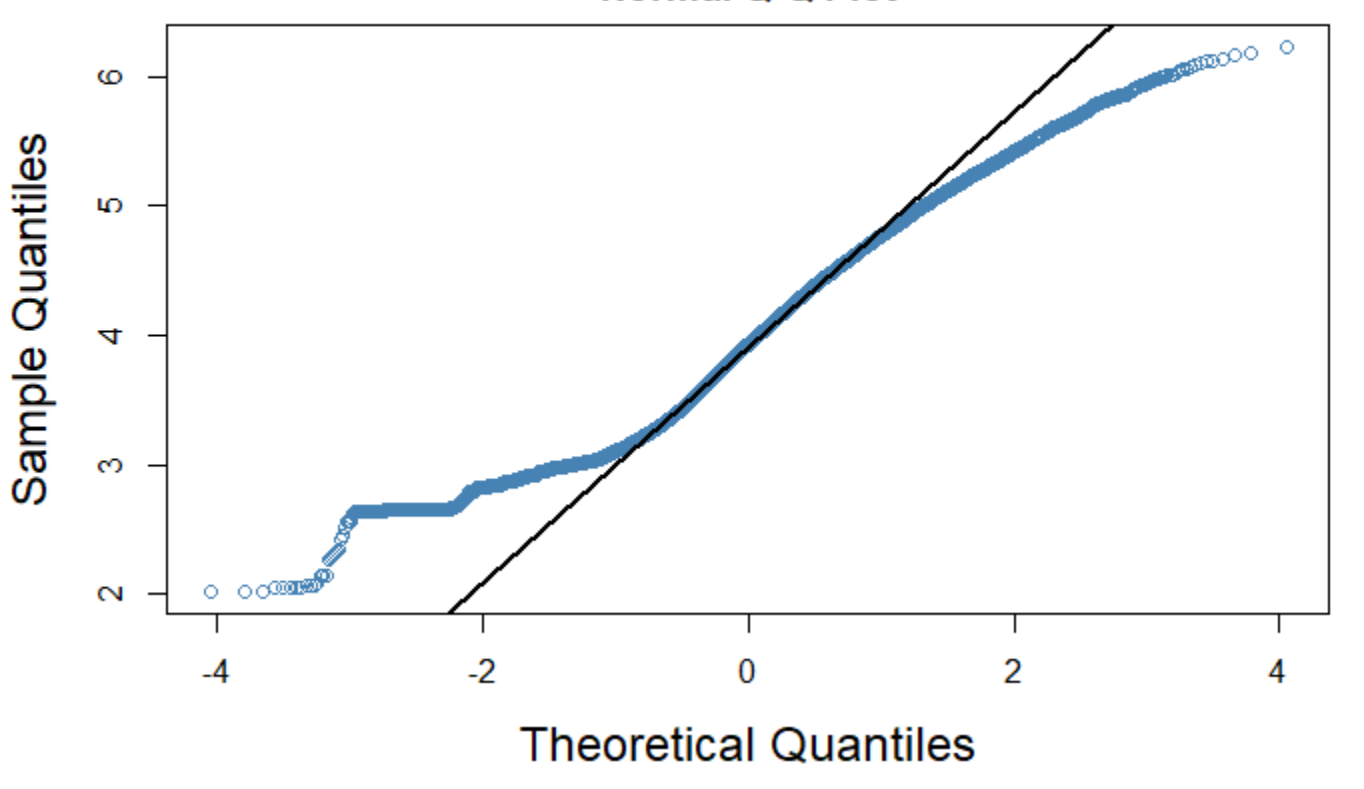


**Figure S4.** Q-Q plot of healthcare expenditures. The log_10_-transformed healthcare expenditures look approximately Normal, with a median of 3.93 ($8,525), and mean± SD of 3.95±0.74 ($36,222).


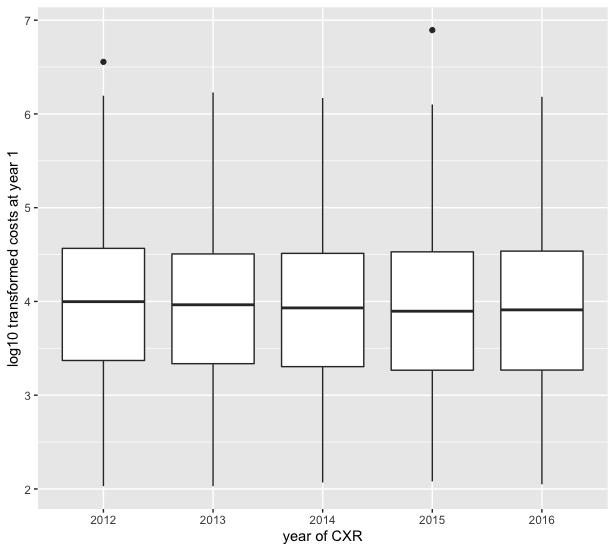


**Figure S5.** Boxplot of log_10_-transformed 1 year expenditure separated by year of chest X-ray.


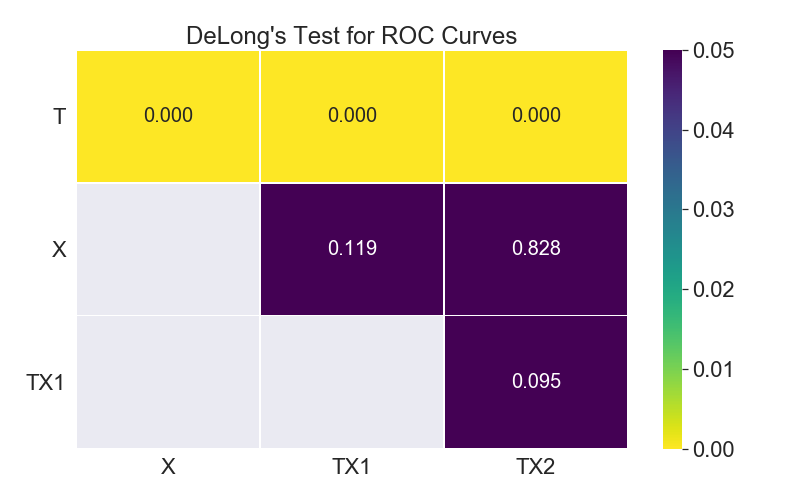


**Figure S6**. Results of pairwise comparison of classification ROC-AUC. P values of pairwise comparison of ROC curves of the 3 classification models. The gray cells are equivalent to the symmetric values on the grid.


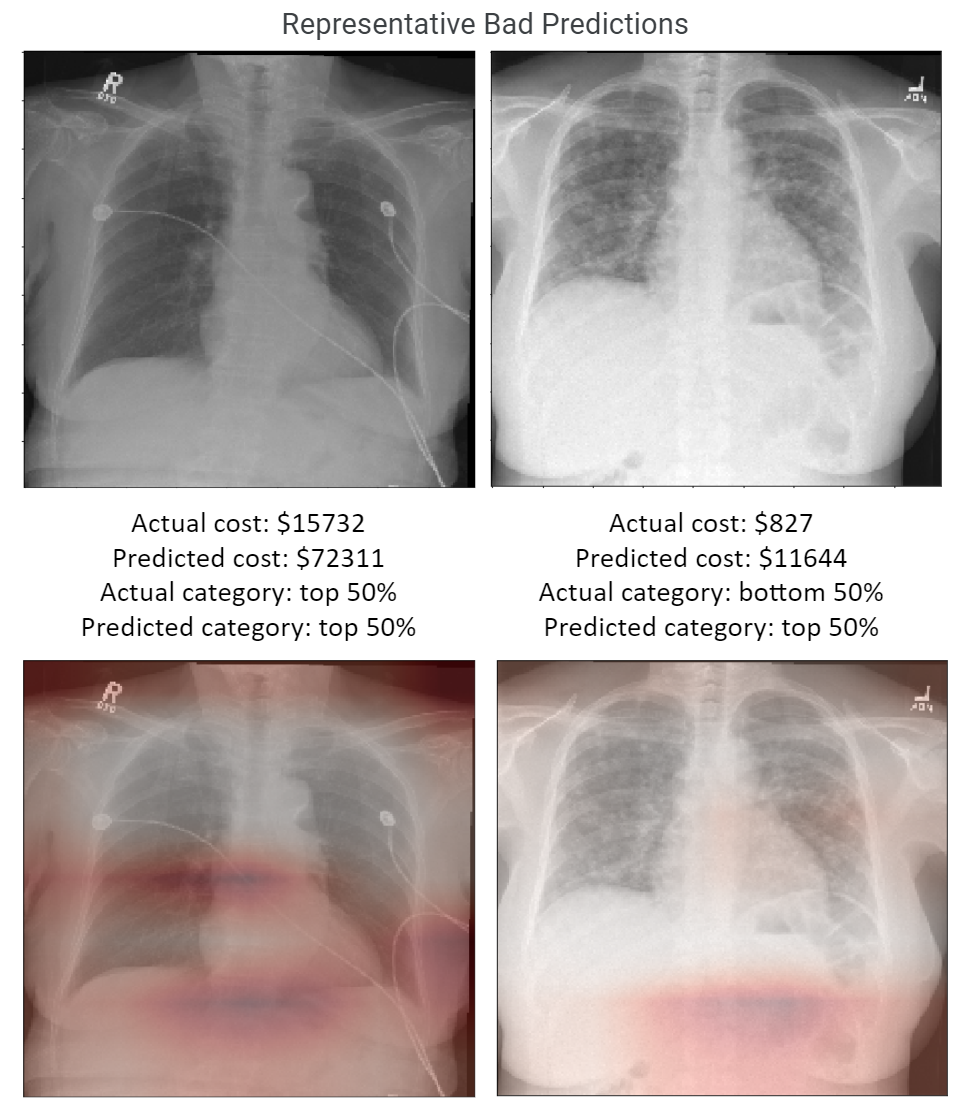


**Figure S7.** Samples of poor predictions. Two examples with poor performance paired with their Grad-CAM maps. The attention maps highlighted the regions that are of most importance to the deep learning model’s decision.

**Table S1**. Excluded Patients vs Included Patients Demographics

| **Characteristics** | **Excluded Count (%)** | **Included Count (%)** |
| --- | --- | --- |
| ***Gender*** (p < 0.0001)* |  |  |
| Female | 308 (30.7) | 10555 (48.3) |
| Male | 696 (69.3) | 11317 (51.7) |
| ***Age*** (p<0.0001)** |  |  |
| ***Cost*** (p<0.0001)** |  |  |
| ***Region*** (p < 0.0001)* |  |  |
| Bay Area | 795 (78.6) | 19125 (87.5) |
| Not Bay Area | 72 (7.1) | 2741 (12.5) |
| Not Assigned | 145 (14.3) | 6 (0.0) |
| ***Race*** (p<0.0001)* |  |  |
| American Indian or Alaska Native | 12 (1.2) | 76 (0.3) |
| Asian | 60 (5.9) | 4720 (21.6) |
| Black or African American | 150 (14.8) | 2547 (11.7) |
| Native Hawaiian or Other Pacific Islander | 13 (1.3) | 494 (2.3) |
| Other | 111 (11) | 3226 (14.7) |
| Unknown/Declined | 102 (10.1) | 751 (3.4) |
| White or Caucasian | 564 (55.7) | 10058 (46) |

Based on the 1004 patients with information on sex and age but no zip code median income.

*p-value is comparing the relationship between inclusion and the corresponding variables using Chi-square test for independence for categorical variables, two-tails at significance level 0.05.

** p-value is comparing the mean between excluded CXR’s and included CXR’s using t-test, two-tails at significance level 0.05. Mean age difference between the two groups is 5.179 year.

**Table S2.** ANOVA Results of Fitting Cost Using Patient Demographics

| **term** | **df** | **sumsq** | **meansq** | **statistic** | ***p*-value** |
| --- | --- | --- | --- | --- | --- |
| sex | 1 | 0.209 | 0.209 | 0.341 | 0.559 |
| factor(age) | 90 | 1150 | 12.7 | 20.8 | 5.3e-313 |
| race | 6 | 85.2 | 14.2 | 23.1 | 2.19e-27 |
| median_income | 1 | 33.8 | 33.8 | 55 | 1.23e-13 |
| sex:factor(age) | 84 | 93.1 | 1.11 | 1.81 | 9.14e-06 |
| sex:race | 6 | 9.58 | 1.6 | 2.6 | 0.016 |
| factor(age):race | 447 | 252 | 0.564 | 0.919 | 0.887 |
| sex:median_income | 1 | 0.53 | 0.53 | 0.864 | 0.353 |
| factor(age):median_income | 83 | 62.2 | 0.75 | 1.22 | 0.0827 |
| race:median_income | 6 | 15.7 | 2.62 | 4.28 | 0.000259 |
| Residuals | 18900 | 11600 | 0.613 |  |  |

**Table S3.** Average Yearly Expenditure From 2012 to 2016

| CXR Year | Average Yearly Cost |
| --- | --- |
| 2012 | 9941.352 |
| 2013 | 9211.016 |
| 2014 | 8528.410 |
| 2015 | 7864.876 |
| 2016 | 8128.197 |

**Table S4.** Comparison of Expenditure Between Patients that Remained vs Dropped Out

| Comparison of patients dropped vs remaining before year | 3 | 5 |
| --- | --- | --- |
| Comparison of expenses during year | 1 | 3 |
| Delta mean log(expenses): dropped out - persisted | -0.030 | -0.095 |
| mean log(expenses): dropped out | 3.940 | 4.121 |
| Mean log(expenses): remained | 3.970 | 4.216 |
| Median: dropped out | 8067 | 14235 |
| Median: remained | 9130 | 17351 |
| p-value (Welch) | 0.001 | 6.7e-09 |
| p-value (Wilcoxon) | 0.0004 | 4.008e-08 |

**Table S5.** Models and the Corresponding Features

| Model | Features | Outcome | Purpose |
| --- | --- | --- | --- |
| AdaBoost Classifier (T: classification) | Sex, age, ZIP code median income | Whether expenditure in top 50% (binary) | Baseline model with only demographic information for comparison |
| ResNet Classifier (X: classification) | Chest X-ray (CXR) images | Whether expenditure in top 50% (binary) | For comparison with other 3 classification models to test the inference capability of deep learning model on only CXR as features. |
| Modified ResNet Classifier Version 1 (TX1: classification) | Sex, age, ZIP code median income, **and** CXR images | Whether expenditure in top 50% (binary) | Testing the combined inference capability of deep learning model on both CXR and demographic features. Model design 1 for combining mix-type inputs. |
| Modified ResNet Classifier Version 2 (TX2: classification) | Sex, age, ZIP code median income, **and** CXR images | Whether expenditure in top 50% (binary) | Testing the combined inference capability of deep learning model on both CXR and demographic features. Model design 2 for combining mix-type inputs. |
| AdaBoost Regressor (T: Regression) | Sex, age, ZIP code median income | Expenditure | Baseline model with only demographic information for comparison |
| ResNet Regressor (X: Regression) | Chest X-ray (CXR) images | Expenditure | For comparison with other 3 classification models to test the inference capability of deep learning model on only CXR as features. |
| Modified ResNet Regressor Version 1 (TX1: Regression) | Sex, age, ZIP code median income, **and** CXR images | Expenditure | Testing the combined inference capability of deep learning model on both CXR and demographic features. Model design 1 for combining mix-type inputs. |
| Modified ResNet Regressor Version 2 (TX2: Regression) | Sex, age, ZIP code median income, **and** CXR images | Expenditure | Testing the combined inference capability of deep learning model on both CXR and demographic features. Model design 2 for combining mix-type inputs. |

**Supplemental Material (eAppendix)**

**Data Processing Procedures**

The 34,743 AP or PA chest radiographs were retrieved as DICOM files. The Python libraries pydicom and gdcm are used to extract the Dicom files. 21,872 scans from 19,524 individuals were successfully matched with patient information and had an identifiable ground truth label of total costs. The extracted images were then randomly separated into 16,399 training images, 2,189 validation images, and 3,284 hold-out test images, while ensuring the absence of the same patients repeating over the three datasets. Data augmentation was performed with horizontal flip and rotation, and intensity inversion. Categorical variables were mapped to binary numbers and cost data was log_10_-transform normalized. Patient zip codes were linked to U.S. Census American Community Survey 2013-2017 household median income data using R package tidycensus. The data workflow is schematically shown in Supplemental eFigure 1.

The processed images were separated into 16,399 training images, 2,189 validation images, and 3,284 test images. We made sure that test set images do not belong to any patients in the training or validation set. During training, sex categories were mapped to numbers (male = 1, and female = 2), cost data is log_10_-transformed normalized and income data is log_10_-transformed.

**Baseline Demographics Model and CXR Only Model**

The regression CXR input only model is a modified Pyroch ResNet18 model. The original 1000 dimension fully connected layer is replaced with a Pytorch Sequential layer consisting of a dropout layer of dropout rate 0.5 and 3 linear layers of output dimensions of 100, 7, and 1 respectively and 2 ReLu layers sandwiched between the linear layers. Mean Squared Error is used as the loss function.

The classification CXR input only model is a modified Pytorch ResNet50 model. The original 1000 dimension fully connected layer is replaced with a Pytorch Sequential layer consisting of a dropout layer of dropout rate 0.5 and 1 linear layers of output dimension 2 and 1 ReLu layers sandwiched between. The CXR images are provided as raw pixels to the model and processed by the convolutional layers. The outputs from the convolutional layers are image-based features for the fully connected neural network layers.

**Training Procedures**

When calculating the Pearson R, the predicted costs and actual costs were log_10_-transformed. The costs were also log_10_-transformed during the training phase to approximate a normal distribution and improve computational efficiency. The 95% confidence interval for ROC-AUC, F1, Pearson R, and Spearman ρ was calculated by bootstrapping with 4000 samples.

Data preprocessing, analyses, and model developments were performed in Jupyter Notebook using Python language version 3.6.8 with packages GDCM, matplotlib, NumPy, pandas, PIL, pydicom, PyTorch, scikit-learn, SciPy, seaborn, and skimage.

**More Exploratory Analysis**

The interaction between age and sex (p=1.14e-5) contributed significantly, with younger women spending more than younger men (till about the age of 45), while older men spending more than older women (past age 75, see Suppl. eFigure 7). Moreover, race ⨉ median income (p=0.00026) and sex ⨉ race (p=0.012) interaction contributed significantly, but not age ⨉ race interaction (p=0.866).

We observed higher median income was associated with lower healthcare expenditures (Pearson R=-0.0537, *p* = 5.4e-14, Spearman ρ=-0.0544, *p* = 2.4e-14, *n*=19,612). As most of the data come from a few postal code areas, we hypothesized that this trend can be spurious due to the difference in income between major postal areas. However, this trend holds even after removing the largest areas that contribute to 50% of the population or have extreme 25 percentile of median income (R=-0.0556, p=2.8e-6, Spearman ρ=-0.0566, *p* = 1.8e-06, *n*=7107).

Statistical analysis was done in R version 3.5.2 using packages: pROC, MEmetrics, boot, dplyr, tidyr, and tidycensus.
